# Supplementary material for: Resolving the full spectrum of human genome variation using Linked-Reads
Source: Genome Res. 2019 Apr;29(4):635–45. doi: 10.1101/gr.234443.118 (PMC6442396; doi:10.1101/gr.234443.118)
Supplement: Supplemental Material [file supp_gr.234443.118_Supplemental_longranger-2.2.zip › longranger-2.2.tar.bz2!/lr2.2-public/lib/stan/cmdstan/stan/doc/api/html/agrad_2rev_2functions_2lmgamma_8hpp_source.html]

Stan: src/stan/agrad/rev/functions/lmgamma.hpp Source File


|  |  |
| --- | --- |
|  | Stan  2.5.0  probability, sampling & optimization |


- Main Page
- Related Pages
- Namespaces
- Classes
- Files

- File List
- File Members

All Classes Namespaces Files Functions Variables Typedefs Enumerations Enumerator Friends Macros Pages

- src
- stan
- agrad
- rev
- functions

lmgamma.hpp

Go to the documentation of this file.

1 #ifndef STAN\_\_AGRAD\_\_REV\_\_FUNCTIONS\_\_LMGAMMA\_HPP

2 #define STAN\_\_AGRAD\_\_REV\_\_FUNCTIONS\_\_LMGAMMA\_HPP

3

4 #include <valarray>

5 #include <stan/agrad/rev/var.hpp>

6 #include <stan/agrad/rev/internal/dv\_vari.hpp>

7 #include <stan/math/constants.hpp>

8 #include <stan/math/functions/lmgamma.hpp>

9 #include <stan/math/functions/digamma.hpp>

10

11 namespace stan {

12  namespace agrad {

13

14  namespace {

15  class lmgamma\_dv\_vari : public op\_dv\_vari {

16  public:

17  lmgamma\_dv\_vari(int a, vari\* bvi) :

18  op\_dv\_vari(stan::math::lmgamma(a, bvi->val\_),a,bvi) {

19  }

20  void chain() {

21  double deriv = 0;

22  for (int i = 1; i < ad\_ + 1; i++)

23  deriv += stan::math::digamma(bvi\_->val\_ + (1.0 - i) / 2.0);

24  bvi\_->adj\_ += adj\_ \* deriv;

25  }

26  };

27  }

28

29  inline var lmgamma(int a, const stan::agrad::var& b) {

30  return var(new lmgamma\_dv\_vari(a, b.vi\_));

31  }

32

33  }

34 }

35 #endif

digamma.hpp

dv\_vari.hpp

lmgamma.hpp

stan::agrad::var::vi\_

vari \* vi\_

Pointer to the implementation of this variable.

**Definition:** var.hpp:40

stan::math::digamma

double digamma(double x)

**Definition:** digamma.hpp:39

var.hpp

stan::agrad::var

Independent (input) and dependent (output) variables for gradients.

**Definition:** var.hpp:27

constants.hpp

stan::agrad::lmgamma

fvar< typename stan::return\_type< T, int >::type > lmgamma(int x1, const fvar< T > &x2)

**Definition:** lmgamma.hpp:16


---

[ Stan Home Page ]

*© 2011–2014,
Stan Development Team.*
